# Genetic and phenotypic analysis of the virulence plasmid of a non-Shigatoxigenic enteroaggregative Escherichia coli O104:H4 outbreak strain

**DOI:** 10.1099/mic.0.001550

**Published:** 2025-03-27

**Authors:** Rachel Whelan, Martyna Cyganek, Charlotte L. Oxley, Benjamin Dickins, Jonathan C. Thomas, Gareth McVicker

**Affiliations:** 1 Department of Biosciences, Nottingham Trent University, Clifton, Nottingham, NG11 8NS, UK

**Keywords:** enteroaggregative, motility, plasmid, post-segregational killing, toxin–antitoxin

## Abstract

Enteroaggregative *Escherichia coli* O104:H4 is best known for causing a worldwide outbreak in 2011 due to the acquisition of a Shiga-like toxin alongside traditional enteroaggregative virulence traits; however, whilst the 2011 outbreak strain has been well studied, the virulence plasmid of O104:H4 has been subjected to far less experimental analysis. In this paper, we analyse the genetic and phenotypic contribution of the pAA virulence plasmid to a non-Shigatoxigenic O104:H4 strain (1070/13) that was nonetheless implicated in a substantial UK outbreak in 2013. We find that pAA_1070_ is 99.95% identical across 88% of the plasmid sequence to pTY2 from the 2011 outbreak strain and has a copy number of ~2–3 plasmid molecules per chromosome. We demonstrate that pAA_1070_ carries a functional CcdAB plasmid addiction system that only marginally impacts its stability under the conditions tested. None of the other toxin–antitoxin systems encoded by the plasmid appear to be functional, though we note a surprisingly high stability of the plasmid *in vitro* regardless. We demonstrate the expected contribution of pAA_1070_ to intestinal cell adhesion but find that it does not contribute to biofilm formation. When assessing the impact of pAA_1070_ on motility, we discovered a region of the O104:H4 chromosome that can be excised, abolishing motility via truncation of the *fliR* gene. Ultimately, this work demonstrates the importance of mobile genetic elements to enteroaggregative *E. coli* as a pathovar in its own right and highlights the complexity but necessity of experimentally characterizing genuine outbreak strains rather than laboratory strains in order to understand virulence phenotypes.

**Impact Statement**

Enteroaggregative *Escherichia coli* causes severe foodborne illness in humans. One serotype, O104:H4, caused a worldwide outbreak in 2011 that infected almost 4,000 people and killed dozens. The 2011 strain contained two key virulence factors: the Shiga toxin encoded by a bacteriophage and a plasmid that encodes aggregative adherence fimbriae for adhesion to host cell surfaces. Whilst the Shiga toxin and fimbriae have been studied extensively, the role of the plasmid as a whole remains unclear. In this study, we characterize the plasmid of a non-Shigatoxigenic O104:H4 outbreak strain and find that it contributes to host cell adherence, as expected, and that it encodes a functional CcdAB toxin–antitoxin stability system. We also demonstrate that part of the strain’s chromosome can be spontaneously lost during growth in the laboratory, converting the organism from highly motile to non-motile and suggesting a future evolutionary route for the bacterium, as has occurred in similar enteric pathogens such as *Shigella*. In summary, this work contributes to our knowledge of often-neglected enteroaggregative *E. coli* as an important pathovar and highlights the necessity of experimentally characterizing genuine outbreak strains rather than ‘domesticated’ laboratory strains.

## Data Summary

The authors confirm that all supporting data, code and protocols have been provided within the article or through supplementary data files. Hybrid whole-genome sequence data are available for strains 1070/13 (accession numbers CP171474, CP171475 and CP171476) and 1070/13 pAA^–^ (accession numbers CP171472 and CP171473). Sequencing data for 1070/13 produced by Dallman *et al*. [1] are available from the European Nucleotide Archive: sample accession SAMN02730196.

## Introduction

*Escherichia coli* is a common enteric bacterium in mammals. Due to its propensity for mobile genetic element (MGE) acquisition, commensal or otherwise avirulent *E. coli* can be converted into pathogenic forms known as pathovars, defined by their mode of virulence in human disease [2]. One such diarrhoeagenic pathovar, enteroaggregative *E. coli* (EAEC), is phenotypically characterized by a ‘stacked-brick’ adherence pattern on intestinal epithelial cells [3]. This phenotype is thought to result from the production of aggregative adherence fimbriae (AAF) typically encoded on a large (≥70 kb) aggregative adherence plasmid (pAA).

In mid-2011, EAEC serotype O104:H4 made headlines after infecting almost 4,000 people and killing over 50 otherwise-healthy adults [4]. The atypical outbreak variant was found to be a hybrid pathovar, carrying both the expected EAEC virulence genes on its pAA variant, named pTY2 in sequenced strain TY2482 [5,6], and a gene encoding Shiga-like toxin variant Stx2a on a prophage [4,7]. The carriage of both traits likely dramatically enhanced the strain’s virulence relative to both non-enteroaggregative enterohaemorrhagic *E. coli* (EHEC) and non-enterohaemorrhagic EAEC. Whilst Shiga-like toxins are highly studied virulence factors of EHEC, the virulence plasmid of EAEC is far less well characterized. AAF are found in five different forms [8,12], and the pAA plasmids that encode them are similarly varied. For example, the 2011 outbreak plasmid pTY2 is ~75 kb in size and encodes AAF/I, whereas the prototypical EAEC strain 042 (serotype O44:H18) carries a plasmid of ~113 kb that encodes AAF/II, and another strain of O104:H4, EAEC 55989, carries a 72 kb plasmid encoding AAF/III [13,14]. Some sequenced pAA variants have reached over 140 kb in size (e.g. pNORD58_1; accession CP139842.1), and some contain a range of accessory antimicrobial resistance genes (e.g. pLAO63; accession OP242283.1 [15]). After a thorough recent analysis, Boisen *et al.* [16] proposed that EAEC be characterized genetically by the presence of both AAF and the plasmid-encoded master regulator, AggR. Other genes such as those encoding the anti-aggregation protein dispersin and its related transporters are also highly represented amongst EAEC strains [17,20].

Given their size, low copy number and propensity to encode complex protein-based macromolecules (e.g. specialized fimbriae or secretion systems), enteric virulence plasmids are often associated with a growth burden and can be lost or degraded when their specific function is not required, such as during growth outside the pathogen’s preferred niche or in laboratory culture. To counter this, many such plasmids encode maintenance systems or induce compensatory mutations in the bacterial host [21,22]. Toxin–antitoxin (TA) loci are plasmid maintenance systems comprised of genes encoding a stable toxin protein and unstable antitoxin, which can itself be either a protein or RNA molecule. In type II TA systems, both toxin and antitoxin are proteins, and the antitoxin functions by directly binding to and sequestering the toxin [23]. Type II antitoxins are rapidly degraded by cellular proteases such as Lon [24,25]. After bacterial cell division, the loss of a plasmid carrying TA genes results in toxin activation and bacterial growth arrest or death, as the antitoxin is degraded and not replaced; hence, a growing population must retain the plasmid to avoid this post-segregational killing (PSK). Two well-characterized type II TA systems are *ccdAB*, encoding DNA gyrase poison CcdB and its antidote CcdA [26], and *vapBC*, encoding tRNA-cleaving toxin VapC and its antidote VapB [25,27]. Both *ccdAB* and *vapBC* are common in *E. coli* and *Shigella* virulence plasmids, though their stability functions vary [28,31]. There is little to no information regarding EAEC TA systems in the literature, though we have noted the existence of genes putatively encoding a range of TA systems such as CcdAB, VapBC, PemIK, ParDE, RelBE and Hok/Sok on sequenced pAA variants [1,32, 33].

In 2013, a multi-pathogen foodborne disease outbreak in Newcastle, UK [1], was found to be predominantly caused by EAEC strains carrying a wide range of pAA plasmids. Whilst these isolates were all Shiga toxin negative, amongst the strains were O104:H4 isolates carrying a form of pAA almost identical to pTY2 from the 2011 outbreak [1]. The 2013 Newcastle strains can therefore be used as convenient model organisms to study the virulence and maintenance functions of this particular pAA variant.

Given their role in pathoadaption, knowledge of the impact and stability of *E. coli* MGEs is essential to understand how the organism causes disease and how it is able to amass multiple MGEs in order to form hybrid pathovars. In this study, we characterize various virulence functions of pAA in Newcastle outbreak strain 1070/13 and analyse the role of CcdAB encoded on this plasmid. We also identify a region within the chromosome of the organism that is able to spontaneously excise and disable motility in the strain, perhaps suggesting a future evolutionary step for the organism.

## Methods

### Bacterial strains and growth conditions

Bacterial strains (Table S1, available in the online Supplementary Material) were grown in lysogeny broth (LB; Sigma-Aldrich, UK) or on solid l-agar [LB+1.5 % (w/v) agar (Sigma-Aldrich)] supplemented with relevant antibiotics unless otherwise stated. Bacteria were incubated at 37 °C (with aeration for liquid cultures) unless otherwise stated. Antibiotics (Sigma-Aldrich) were used at the following final concentrations for selection and plasmid maintenance: neomycin 50 µg ml^−1^, chloramphenicol 30 µg ml^−1^ and ampicillin 100 µg ml^−1^. Sucrose agar for quantification of pSTAB plasmid loss was made according to the following recipe: 10 g l^−1^ tryptone (Sigma-Aldrich), 5 g l^−1^ yeast extract (Fisher Scientific, UK), 10% (w/v) sucrose (Sigma-Aldrich) and 1.5% (w/v) agar. Where required for the growth of auxotrophs, diaminopimelic acid (DAP; Sigma-Aldrich) was used at 0.3 mM final concentration. PBS (Sigma-Aldrich) was used to wash and dilute bacterial cells. Minimal media were prepared from 5× M9 salts (Sigma-Aldrich), supplemented with 1 mM MgSO_4_ (Sigma-Aldrich) and either 0.2% (v/v) glycerol (Sigma-Aldrich) or 0.2% (w/v) casamino acids (Fisher Scientific). Yeast extract casamino acids (YESCA) medium containing Congo red was prepared as follows: 10 g l^−1^ casamino acids (Merck), 1 g l^−1^ yeast extract and 2% (w/v) agar, supplemented with 0.005% (w/v) Congo red (Sigma) and 0.001% (w/v) Coomassie brilliant blue G (Sigma) after autoclaving.

### DNA manipulation and cloning

Plasmids (Table S2) were purified from overnight bacterial cultures using the Monarch Plasmid Miniprep Kit (New England Biolabs, UK). PCR was carried out using Q5 high-fidelity polymerase (New England Biolabs) for cloning or AppTaq RedMix (Appleton Woods, UK) for colony screening. Oligonucleotide primers (Table S3) with and without modifications were purchased from Eurofins Genomics (Germany). Amplicons were purified using the Wizard SV Gel and PCR Clean-Up System (Promega, UK). Genomic DNA was purified using the Wizard® Genomic DNA Purification Kit (Promega). Plasmids were assembled using NEBuilder HiFi DNA Assembly (New England Biolabs).

### Plasmid curing

To remove the pAA plasmid from strain 1070/13, a repeated heat shock protocol was used. An overnight bacterial culture was diluted to OD_600nm_=1 in 1 ml room-temperature LB, from which 100 µl was taken and added to 5 ml LB pre-warmed to 42 °C. The sample was incubated at 42 °C for 20 min. Following this, 100 µl culture was transferred into 5 ml room-temperature LB and grown overnight at 37 °C with aeration, after which the dilution and 42 °C incubation steps were repeated as above. Finally, the culture was serially diluted, and 5 µl of each dilution in triplicate was spotted onto l-agar and grown overnight at 37 °C. Colonies were analysed by PCR to verify plasmid loss.

### Conjugative mutagenesis

Deletion of the *ccdAB* and *aggR* genes from pAA_1070_ was achieved through conjugative mutagenesis, since other methods (e.g. lambda red recombineering) failed to produce satisfactory results in *E. coli* 1070/13. Briefly, isothermal DNA assembly was used to produce variants of pCONJ5K [34] containing a chloramphenicol resistance cassette flanked by pAA DNA sequences surrounding either *ccdAB* or *aggR. E. coli* MFD*pir* Δ*hsdR* carrying either plasmid variant was used as a donor strain for conjugation into recipient *E. coli* 1070/13. Donor strains were grown overnight in LB+DAP+chloramphenicol. The recipient strain was grown overnight in LB. From each, 1 ml culture was pelleted (16,000 ***g*** for 2 min) and washed with an equal volume of PBS. Donor (20 µl) was mixed with 20 µl recipient, and the total volume was spotted onto L-agar+DAP. When dry, the plate was incubated at 37 °C with aeration for 4 h. Following incubation, bacteria were resuspended in 1 ml LB, and one-tenth of the volume was plated onto l-agar containing chloramphenicol for incubation overnight at 37 °C. Colony PCR was performed to check for the first crossover event, and positive colonies were grown overnight in LB+chloramphenicol. From this, 1 ml culture was pelleted and washed three times in LB, then a further 4 ml LB was added and the culture was grown for 4 h at 37 °C with aeration. From the incubated culture, 5 µl was plated onto sucrose agar and incubated overnight to obtain second crossover mutants (i.e. plasmid backbone excision). Final mutants were screened by colony PCR and Sanger sequencing.

### DNA sequencing and analysis

PCR amplicon sequencing for verification of clones and mutants was performed by Source BioScience (UK). Whole-genome hybrid Illumina/Nanopore sequencing of strains 1070/13 and 1070/13 pAA^–^ was performed by MicrobesNG (UK). MicrobesNG prepared Illumina sequencing libraries using the Nextera XT Library Prep kit (Illumina, UK) prior to sequencing on an Illumina NovaSeq 6000, for 2×250 bp paired-end reads. Adapters were trimmed from reads using Trimmomatic v0.30 with a sliding window quality cut-off of Q15 [35] and a minimum read length of 36 bp. Nanopore sequencing libraries were prepared using the Oxford Nanopore SQK-LSK109 kit with Native Barcoding kits EXP-NBD104/114, prior to loading on a GridION with a FLO-MIN106 (R.9.4.1) flow cell. Raw data were basecalled using the model 2021-05-17_dna_r9.4.1_minion_384_d37a2ab9.

Hybrid genomes were assembled in-house according to Eladawy *et al.* [36]. Briefly, end and middle adapters were trimmed from Nanopore sequencing reads using Porechop v0.2.4 (https://github.com/rrwick/Porechop) with thresholds of 95% and 85%, respectively. Filtlong v0.2.1 was used to remove short reads, <1 kbp. Overlapping reads were assembled into complete circular molecules using Flye v2.9.2 [37], prior to being polished with both Nanopore and Illumina reads. Assembled contigs were first polished with Nanopore reads, using four iterations of Racon v1.5.0 [38] and one iteration of Medaka v1.8.0. Subsequently, Illumina reads were used to polish the resulting sequences, using Polypolish v0.5.0 [39], POLCA from the MaSuRCA v4.1.0 package [40] and Nextpolish v1.4.1 [41]. Visual alignment was produced using clinker gene cluster comparison [42] (https://cagecat.bioinformatics.nl/).

### Toxicity assay

To test the effect of toxins and antitoxins cloned onto pBAD33 and pGM101_neo_ derivatives, respectively, the following protocol was used as previously described [30]. Strains carrying test plasmids or empty vector controls were grown overnight in LB+antibiotics+0.2% (w/v) glucose (Sigma-Aldrich) and then subcultured to a starting OD at 600 nm (OD_600nm_) of 0.01 in fresh media. Upon re-growing to OD_600nm_=0.1–0.2, bacterial cells were pelleted by centrifugation at 4,000 ***g*** for 10 min, and the supernatant was discarded. Cells were resuspended in pre-warmed LB+antibiotics+1 % (w/v) arabinose (Sigma-Aldrich). Immediately and at timed intervals thereafter, cultures were serially diluted and plated onto l-agar containing antibiotics+0.2% (w/v) glucose. Plates were incubated at 37 °C overnight to observe bacterial viability.

### Human cell culture and adhesion assay

HT29 cells were grown in Dulbecco’s modified Eagle’s medium (DMEM) with high glucose (Sigma-Aldrich), 10% (v/v) FBS (Fisher Scientific), 2% (v/v) HEPES (v/v) (Sigma-Aldrich) and 10 µg ml^−1^ PenStrep (Fisher Scientific) in a T75 flask. Caco-2 cells were grown in DMEM and 10% (v/v) FBS. Both cell lines were grown in a humidified incubator with 5% CO_2_ at 37 °C. Cells were harvested after reaching ~80% confluency by adding 2 ml TrypLE Express (Fisher Scientific). Cells were seeded at 10^5^ cells per well in a 24-well plate the day before infection. All work on HT29 cells was carried out at the University of Oxford. Experiments using Caco-2 cells were carried out at Nottingham Trent University.

For the adhesion assay, bacteria were grown overnight and then subcultured in 10 ml LB to OD_600nm_=0.01 and re-grown to OD_600nm_=0.6. Cultures were then centrifuged at 4,000 ***g*** for 5 min, and the pellet was resuspended in PBS before a further 2-min centrifugation. The pellet was then resuspended in pre-warmed DMEM to the volume harvested (taking into account the culture removed for OD measurements). From this, the starting inoculum was serially diluted and plated onto l-agar for confirmation. The 24-well plate containing a confluent monolayer of eukaryotic cells was checked via microscopy before the growth media were removed from each well. Wells were then inoculated with 1 ml DMEM containing 10^7^ bacterial cells per well (m.o.i=100) in triplicate. An additional well per strain that was not seeded with eukaryotic cells was inoculated with bacteria to account for any adherence of bacteria to the surface of the well itself, the result of which was subtracted from test wells. The 24-well plate containing the infected eukaryotic cells was then incubated at 37 °C, 5% CO_2_ for either 30 min or 3 h. Following incubation, media were removed from all wells, and wells were washed thrice with 1 ml PBS to remove any non-adhered bacteria. One millilitre DMEM containing 1 =% (v/v) Triton X-100 (Promega) was then added to each well with vigorous pipetting to detach and disrupt cells from the well surface. Samples from each well were then diluted and plated on l-agar and incubated at 37 °C overnight. Results were normalized to the starting inoculum.

### Biofilm assay

Overnight cultures were diluted to OD_600nm_=0.5 in PBS. LB (900 µl) was added to each well in a 24-well plate to which 100 µl diluted culture was inoculated in triplicate. Control (blank) wells contained LB only. The 24-well plate was incubated at 37 °C for 18 h. The growth medium was carefully removed by tipping and blotting. Wells were then washed with 1 ml PBS by shakiyng at 100 r.p.m. for 5 min. PBS was then removed and the wash was repeated. Each well was stained with 500 µl of 0.1% (w/v) crystal violet dye (Sigma-Aldrich) and was incubated at room temperature for 1 h. Excess dye was then drained, and wells were washed gently with 1 ml PBS and then tipped and blotted. This wash step was then repeated until the PBS remained colourless when added to the wells. To solubilize the dye, 200 µl 70% (v/v) ethanol (Fisher Scientific) was added to each well, and the plate was shaken for 2 min at 100 r.p.m. Subsequently, 100 µl was taken from each well into a 96-well plate, and the OD was measured at 540 nm. Values from the blank wells were subtracted to account for well-only staining.

### Motility assay

To assess motility, a single colony was picked via sterile needle from a streak grown overnight and then used to stab the centre of LB+0.3% (w/v) agar containing 0.01% (w/v) triphenyltetrazolium chloride (Sigma-Aldrich). Plates were incubated at 37 °C for 18 h, and then, motility diameter around the stab point was measured.

### Digital droplet PCR

For digital droplet PCR (ddPCR), the phenol-chloroform method of DNA purification was used. Overnight bacterial culture (600 µl) was added to a phase lock tube. One volume of phenol:chloroform:isoamyl alcohol (25:24:1) (Sigma-Aldrich) was added to the sample and vortexed. The sample was centrifuged at room temperature for 5 min at 16,000 ***g***. The upper aqueous phase was transferred to a fresh tube. Reagents were added to the aqueous phase in the following order: 1 µl glycogen 20 µg µ^−l^ (Sigma-Aldrich), 0.5× sample volume 7.5 M ammonium acetate (Sigma-Aldrich) and 2.5× sample volume 100% ethanol. DNA was precipitated overnight at −20 °C. The sample was then centrifuged at 4 °C for 30 min at 16,000 ***g**.* The supernatant was removed, and 150 µl of 70% (v/v) ethanol was added. The sample was centrifuged at 4 °C for 2 min at 16,000 ***g***, and ethanol was removed. The DNA pellet was dried at room temperature for 10 min before being resuspended in 30 µl of nuclease-free water. The DNA concentration was measured spectrophotometrically.

The QX200 Droplet Digital PCR System and recommended consumables (Bio-Rad, UK) were used for all ddPCR experiments. Primers and probes were designed using Primer3plus [43] with probes modified at 5′ end with fluorophore FAM (fluorescein) for the reference gene and HEX (hexachlorofluorescein) for the plasmid marker and modified at the 3′ end with the quencher BHQ-1 (Eurofins Genomics).

The master mix was made as described in Table S4, and 20 μl was added to each well in the DG8 cartridge, followed by 70 µl QX200 droplet generation oil for probes. The cassette was placed into the DG8 cartridge holder and DG8 gasket was added. Droplets were then formed using the QX200 droplet generator. The resulting droplets were carefully transferred to a ddPCR 96-well plate, and PCR plate pierceable heat seal foil was added and sealed using the PX1 PCR plate sealer. PCR was then carried out using the C1000 Touch thermocycler with 96-deep well reaction module with the following PCR conditions: enzyme activation 95 °C for 10 min, 40 cycles of denaturation at 94 °C for 30 s and annealing/extension at 55–64 °C for 1 min and 1 cycle of enzyme deactivation at 98 °C for 10 min. Results were analysed using QuantaSoft software v1.7.4.0917.

### Statistical analysis

All statistical analysis was carried out in GraphPad Prism [44]. Data were log-transformed prior to analysis by either one- or two-way ANOVA or t-test depending upon the experimental requirements. Where relevant, appropriate post-tests were used to interrogate data further. Individual tests for each experiment are detailed in the figure legends. For plasmid loss assays, statistically significant outliers were identified and removed via the Grubbs test; doing so did not change the overall conclusion of the experiments.

## Results

### pAA has a low plasmid copy number typical of other *E. coli* virulence plasmids

*E. coli* O104:H4 strain 1070/13 is representative of the O104:H4 isolates recovered from a multi-pathogen Newcastle outbreak [1]. Strain 1070/13 underwent Illumina whole-genome sequencing after its isolation by Dallman *et al.* and was found to contain a variant of pAA, hereafter referred to as pAA_1070_. To confirm the plasmid sequence, we carried out hybrid genome sequencing of the isolate and revealed an intact plasmid (accession number CP171475) that is 99.95% identical across 88% of the plasmid sequence to pTY2 from the 2011 outbreak strain TY2482 [5,6]. Differences between pAA_1070_ and pTY2 primarily consist of insertion sequence (IS) elements such as IS*Kpn26*-, IS*Ec43*- and IS*66*-related ORFs in pAA_1070_ that appear as assembly gaps in the pTY2 sequence (Fig. S1). To obtain an approximate plasmid copy number for pAA_1070_, we mapped Dallman *et al.*’s short-read sequence coverage of strain 1070/13 separately to the pTY2 plasmid reference sequence [6] and the *E. coli* K-12 MG1655 chromosome sequence [45]. The former gave a mean read depth of 264.6× and the latter a mean read depth of 100.3×, indicating a plasmid copy number of ~2.6 plasmid molecules per chromosome. This result is expected from prior studies on enteric virulence plasmids [46,49]. Long-read sequencing from our hybrid approach agreed, giving a copy number of 2.48 plasmids per chromosome (calculated from an overall plasmid contig depth of 2.82× to a mean chromosome contig depth of 1.138×).

**Fig. 1.**
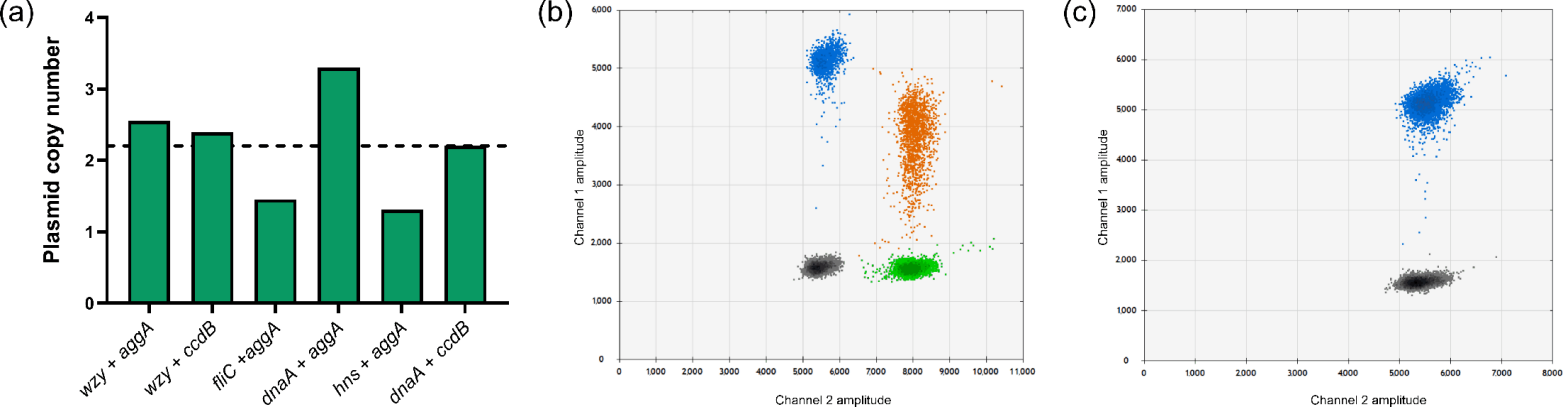
Determination of pAA_1070_ copy number by ddPCR. (a) Plasmid copy number per chromosome, calculated from different probe combinations as indicated. Experiments were performed at an annealing temperature of 61.2 °C with DNA template concentration of 50 ng µl^−1^. Probes targeting chromosomal genes (*wzy*, *fliC*, *dnaA* and *hns*) are labelled with FAM. Probes targeting plasmid genes (*aggA* and *ccdB*) are labelled with HEX. No template controls showed double-negative results for all probe combinations (not quantified on graph). Dotted line: mean pAA_1070_ copy number calculated from all probe combinations. (b, c) Representative 2D amplitude plots of ddPCR assay using *wzy* and *aggA* probes and template DNA from either (b) 1070/13 or (c) 1070/13 pAA^–^. Grey dots, double-negative droplets; blue dots, FAM-positive droplets (*wzy* only); green dots, HEX-positive droplets (*aggA* only); orange dots, double-positive droplets (*wzy* and *aggA*).

To confirm this value experimentally, we carried out ddPCR to determine the absolute DNA quantities of a range of plasmid genes relative to chromosome genes in *E. coli* 1070/13 grown to stationary phase in LB at 37 °C. Results (Fig. 1a) show an estimated plasmid copy number of between ~1.5 and 3.3 depending upon probe combination. The mean of 2.2 plasmid molecules per chromosome was in general agreement with the copy number extrapolated from sequencing data. As a control for the ddPCR assay, we also produced a pAA-cured variant of strain 1070/13. Contrasting the apparent instability of EAEC plasmids reported *in vivo* [50], pAA_1070_ *in vitro* was remarkably stable relative to the virulence plasmids of organisms such as *Shigella* [30], and curing required multiple heat-shock treatments. The resulting strain was designated 1070/13 pAA^–^ and was negative for plasmid probe fluorescence in ddPCR assays, whereas the WT remained positive (Fig. 1b, c).

### pAA carries at least one functional TA system

Bioinformatic analysis of pAA_1070_ revealed the presence of multiple putative TA genes. Immediately adjacent to the FII origin of replication are two operons putatively encoding RelE/ParE-like toxins and their cognate antitoxins. In addition to this, a second TA-related locus ~5–7 kb from the origin contains two divergently transcribed TA operons putatively encoding CcdAB and VapBC. Interestingly, in addition to the expected ATG start codons, BASys annotation [51] predicted alternative upstream start codons associated with putative Shine-Dalgarno sequences for both toxin genes (*ccdB*: TTG and *vapC*: GTG). We noted that VapC includes a specific active site residue (N107, counted from the GTG start codon) that may render the toxin nonfunctional [52].

In order to analyse the roles of these systems, we first cloned the individual toxin genes into an arabinose-inducible expression vector, pBAD33 [53], and assessed the resulting protein products for toxicity in *E. coli* DH5*α*. Alternative start codons are linked to translation under stress [54,56], so we included both variants of each gene in our initial toxicity screen, using the nomenclature CcdB_101_/CcdB_110_ and VapC_133_/VapC_142_ referring to the toxin length in aa. We surmised that, regardless of whether or not the alternative start codons were functional, the longer-length constructs would give the best chance of observing toxicity (barring post-transcriptional control). Similarly, the shorter-length constructs could reveal any requirement for an extended N-terminal sequence.

**Fig. 2.**
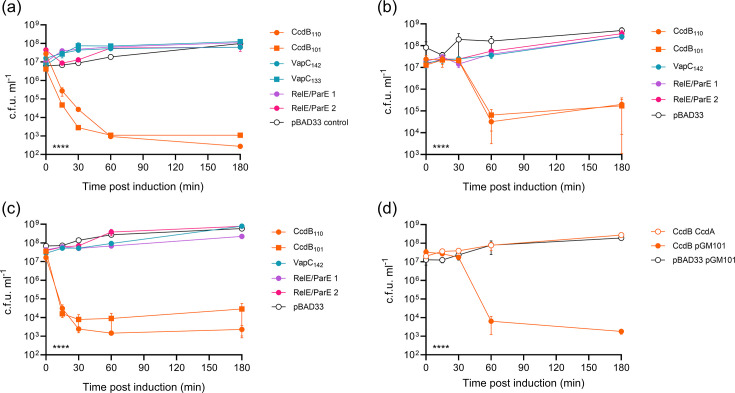
Characterization of TA systems on pAA_1070_. Mean bacterial viability after induction of toxin production from pBAD33 (a) in *E. coli* DH5*α* grown in LB; (b) in *E. coli* 1070/13 pAA^–^ grown in LB, (c) in *E. coli* 1070/13 pAA^–^ grown in M9 with casamino acids and (d) in *E. coli* 1070/13 pAA^–^ grown in LB in the presence/absence of cognate antitoxin produced from pGM101_neo_. Empty vector controls in each case are indicated as pBAD33 and/or pGM101. Error bars show sem. Statistical analysis by two-way ANOVA with Tukey’s multiple comparison tests: indicator in the lower left shows the overall effect of strain in each experiment; ****P<0.0001 (from *n*=3 biological replicates).

Results (Fig. 2a) showed no significant toxicity 180 min post-induction of either VapC or the two RelE/ParE-like toxins (*P*≥0.285 compared to the empty vector control), whereas both CcdB variants were significantly toxic (*P*<0.0001). Interestingly, there was also a minor but significant difference between the effect of the two CcdB variants when compared to one another at 30 min (*P*=0.0024) and 180 min (*P*=0.0178) post-induction. However, as this effect did not persist between *E. coli* strains (see below) and we hence did not verify the proteins produced by our constructs, we do not intend to suggest here that this result is due to the production of an alternate protein variant per se.

To confirm toxicity in the native host strain in the absence of any antitoxin interference from the native pAA plasmid, 1070/13 pAA^–^ was used in a toxicity assay under the same experimental conditions as *E. coli* DH5*α* (Fig. 2b). Due to the potential difference between putative CcdB variants seen in DH5*α*, we included both CcdB_101_ and CcdB_110_ in this experiment, whereas we included only the longer-length VapC_142_ clone. The production of either CcdB variant again significantly reduced cell viability, with the most dramatic effect at 60 min post-induction (*P*<0.02 relative to the empty vector control). In this strain background, there was no significant difference between the two CcdB variants (*P*≥0.96 throughout the assay). As in DH5*α*, there was no significant toxicity observed when expressing any of the non-*ccdB* genes (*P*>0.57).

Suspecting that the effect of the TA systems might be impacted by growth media, we also tested the toxicity of the above constructs during growth in M9 minimal media supplemented with casamino acids (Fig. 2c). Results were similar to those observed during growth in LB, with a significant effect of both CcdB variants on viability (most pronounced at 60 min post-induction, *P*<0.03). We also noted that both VapC and one of the RelE/ParE variants showed significant toxicity at this timepoint (*P*<0.045), but the effect was very minor (a decrease in viability of less than 10-fold for each compared to more than 1,000-fold for CcdB), so this was not pursued further. As in LB, there was no significant difference between the CcdB variants throughout the assay (*P*>0.39).

To verify that CcdAB functions as a cognate TA pair on pAA_1070_, the *ccdA* gene was cloned onto a second vector, pGM101_neo_, under the control of its native promoter to allow for induction of gene expression via conditional cooperativity [57]. pGM101_neo_ was constructed as a neomycin-resistant derivative of the promoterless, pBAD33-compatible vector pGM101 [30], used to ensure selection in the ampicillin-resistant 1070/13 strain. A toxicity assay was then conducted in the 1070/13 pAA^–^ host background using CcdB_110_ as the representative toxin construct. As expected, the expression of *ccdB* from pBAD33 in the presence of the *ccdA*-containing vector no longer resulted in the loss of bacterial cell viability (Fig. 2d; *P*>0.23 vs. empty vector control throughout). These results imply that the *ccdAB* operon on pAA_1070_ encodes a classical TA system.

Lastly, to investigate the mode of action of CcdB from pAA_1070_, we tested the toxicity of our pBAD33 constructs in two commercial CcdB-resistant strains: *ccdB* Survival 2 T1^R^ (Invitrogen; resistance genotype not known) and DB3.1 (originally from Invitrogen but no longer available; known to harbour the *gyrA462* allele). The *Shigella flexneri* M90T invasion plasmid (pINV) variant of CcdB [30] was included as a control. Results showed that whilst all toxin variants reduced the viability of *ccdB* survival cells relative to the empty vector (Fig. S2A, *P* ≤ 0.0012 at 180 min post-induction), there was no toxicity in DB3.1 (Fig. S2B, *P*>0.14 at 180 min post-induction). This suggests that CcdB from pAA_1070_ targets DNA gyrase, as expected.

In order to assess the impact of the CcdAB module on plasmid maintenance, we replaced the *ccdB* gene with a chloramphenicol resistance cassette, rendering the TA locus non-functional. Counterselectable markers (e.g. *sacB*) have been used in TA research to successfully quantify very small levels of plasmid loss [30], but we were unable to generate such a pAA mutant, for reasons unknown. We therefore relied initially upon quantification of plasmid loss via PCR and/or plating to agar with and without chloramphenicol. Our experiments revealed no measurable destabilization of pAA within the detection limit of our experiments, regardless of the presence or absence of *ccdAB* (<1% plasmid loss for both the WT and Δ*ccdB* strain).

**Fig. 3.**
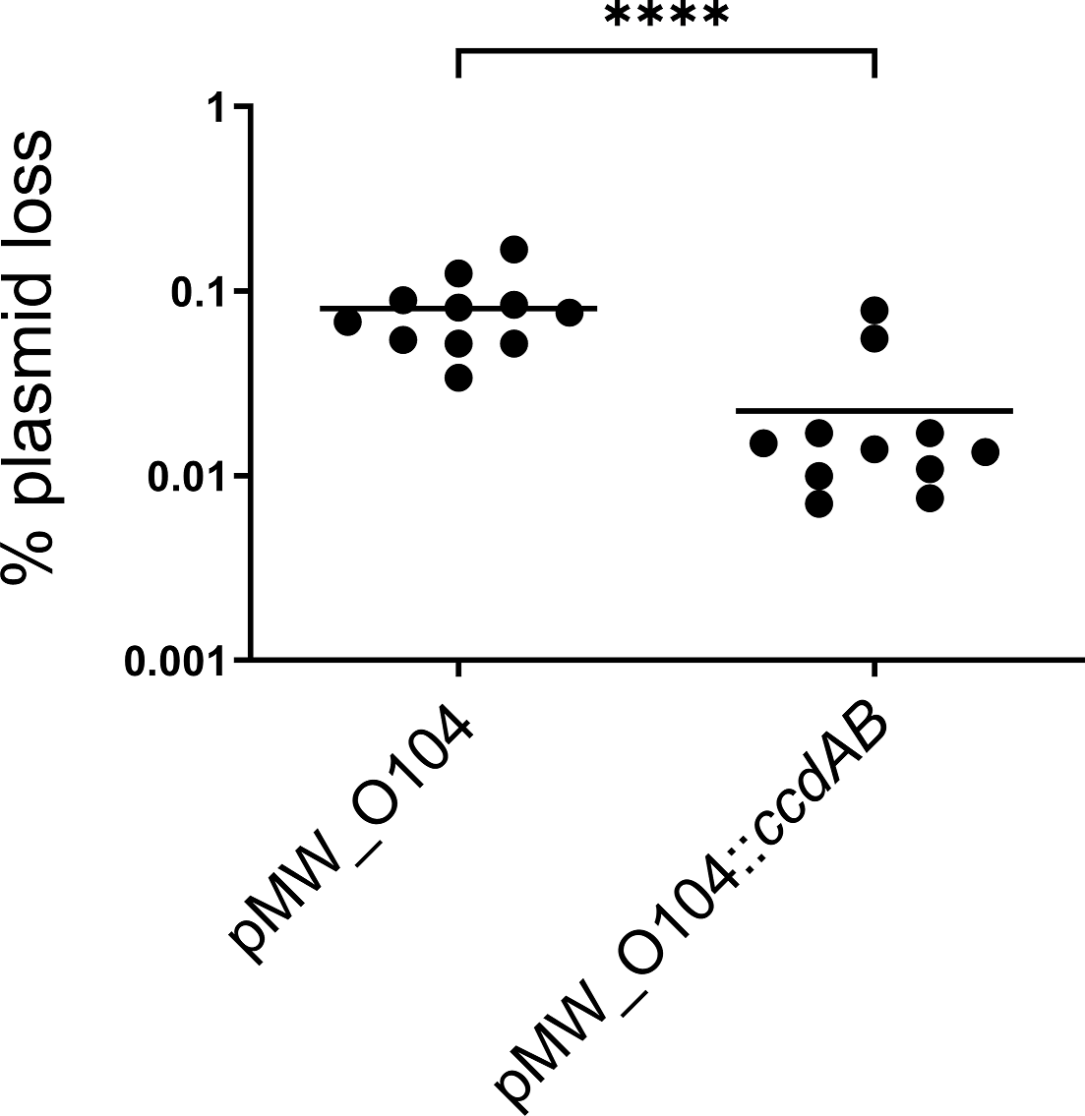
Effect of CcdAB on a pSTAB test vector containing the natural FII/FIB dual replicon region of pAA1070. Plasmid stability in *E. coli* NEB 10-beta was determined by plating onto neomycin (plasmid positive) or sucrose (plasmid negative) in accordance with the previous work [30,60]. Each point shows plasmid loss as a percentage of the total viable cell population within a single biological replicate (one independent colony grown on L-agar for 20–25 generations at 37 °C). Solid lines show the mean. Data combined from multiple independent experiments. Statistical analysis by t-test: ****P<0.0001.

Given the low detection limit of the above experiments to quantify plasmid loss, we constructed a stability test vector (‘pSTAB’) with and without the *ccdAB* locus from pAA_1070_. pSTAB vectors contain a selectable neomycin resistance marker as well as a counterselectable *sacB* gene [58], improving the detection limit in experiments to ~0.0001% plasmid loss [59,60]. These vectors rely upon the native virulence plasmid replicon for replication and copy number control; interestingly, we were only able to obtain a successful pSTAB clone (designated pMW_O104) by including the entirety of the 6,530 bp dual FII/FIB replicon from pAA_1070_, unlike a previous *Shigella* pINV study in which the 2,600 bp FII replicon alone was sufficient [60]. Quantifying pMW_O104 and pMW_O104::*ccdAB* plasmid loss over ~20–25 generations of growth, we determined that the CcdAB system provides a minor but statistically significant improvement in plasmid stability (Fig. 3, *P*<0.0001).

### pAA_1070_ influences adhesion to host cells but not biofilm formation

EAEC plasmid research has typically been carried out in TY2482 or other strains from the 2011 outbreak, which contain a Shigatoxigenic phage [4,7, 50], or in strains with different variants of the plasmid, e.g. prototypical EAEC strain 042 [14,61,63]. In order to elucidate the phenotypic role of pAA in a pathogenic strain closely related to TY2482 but lacking the Shiga toxin, 1070/13 pAA^–^ and its parent strain were subjected to a range of tests to assess any change in growth rate, biofilm formation and adherence to intestinal cells *in vitro*.

**Fig. 4.**
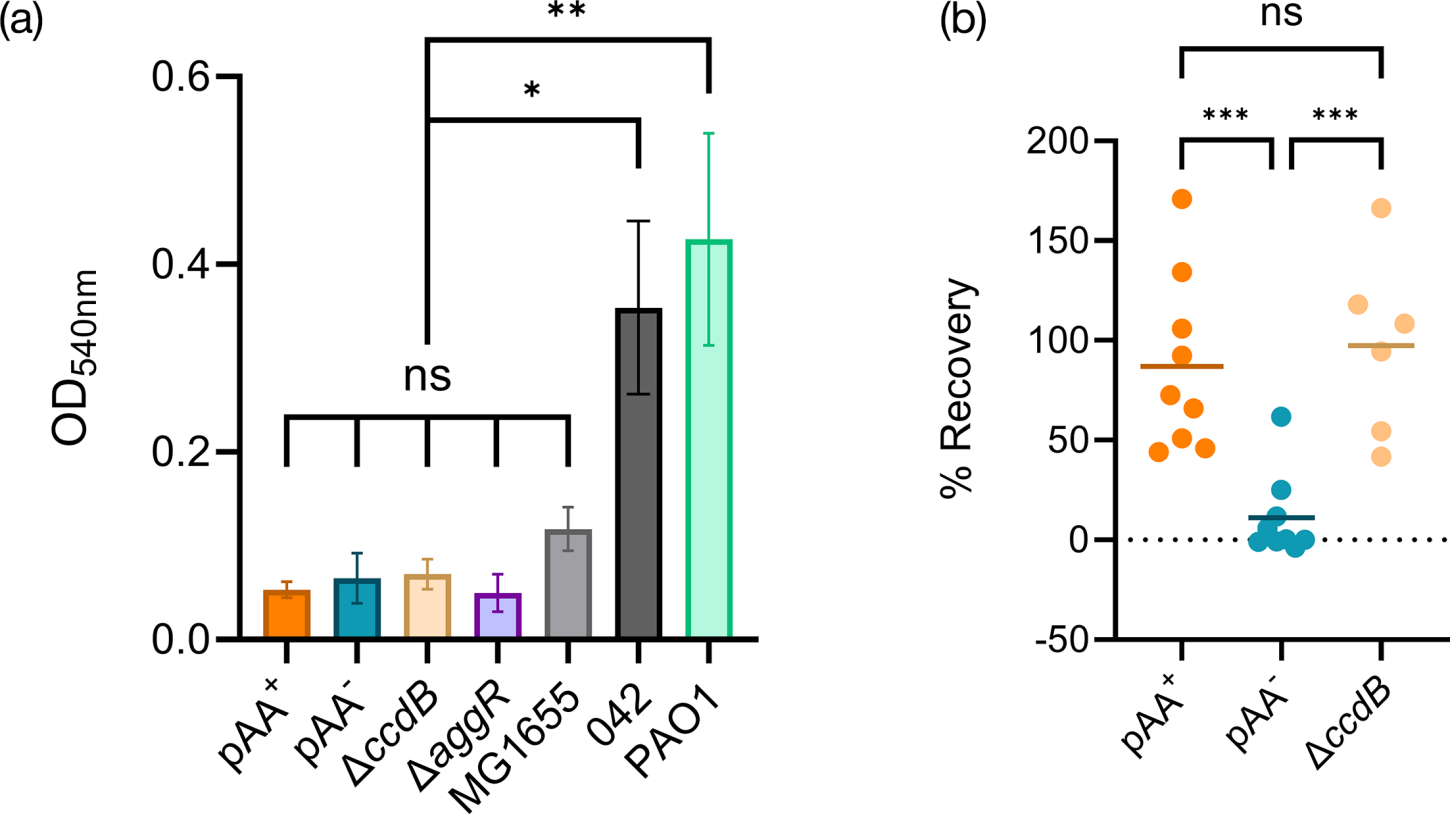
Effect of pAA on virulence-related phenotypes of O104:H4 strain 1070/13. (a) Mean biofilm production after 24-h growth in LB at 37 °C, assayed via crystal violet staining. Controls: EAEC 042 and *P. aeruginosa* PAO1. Error bars show sem. (b) Per cent recovery of bacterial cells (normalized to inoculum) after incubation for 180 min with Caco-2 cells. Each point is one biological replicate. Solid lines show the mean. Statistical analysis by one-way ANOVA with Tukey’s multiple comparison tests: ns, not significant (*P*≥0.05); **P<0.01; ***P <0.001 (from *n*≥3 biological replicates).

Loss of pAA_1070_ resulted in no significant change in growth rate or growth yield for bacteria grown in either rich medium (LB), minimal medium supplemented with glycerol or minimal medium supplemented with casamino acids (Fig. S3). Similarly, there was poor biofilm production (Fig. 4a) from the strain regardless of whether it contained the plasmid (pAA^+^ vs. pAA^–^, *P*>0.9999). Overall, O104:H4 biofilm production was similar to that of *E. coli* K-12 laboratory strain MG1655 (all O104:H4 strains tested vs. MG1655, *P*>0.95). Notably, EAEC strain 042 [14] exhibited significantly higher biofilm production than the O104:H4 variants (*P*<0.02). A *Pseudomonas aeruginosa* PAO1 control [64] also showed significantly higher biofilm production (PAO1 vs. O104:H4 strains, *P*<0.003). To qualitatively assess the production of extracellular polymers, we grew strains at 37 °C on YESCA medium containing Congo red dye (Fig. S4). Interestingly, the WT 1070/13 strain took up less dye than the pAA^–^ derivative and was comparable to strain 042.

We next assessed the ability of 1070/13 and 1070/13 pAA^–^ to adhere to human colon epithelial cells *in vitro*. As expected, the loss of pAA_1070_ significantly reduced the organism’s ability to adhere to both Caco-2 cells (Fig. 4b, *P*=0.0008) and HT-29 cells (Fig. S5A, *P*=0.0067), most likely due to the lack of AAF. This is consistent with the previously published work on other EAEC strains [63,65,67], so we did not investigate this particular phenotype further. However, we noted that the human cells co-cultured with WT EAEC varied dramatically in their morphology from those co-cultured with the plasmid-free variant or the non-infected control (Fig. S5B–D). The Δ*ccdB* mutation did not affect biofilm formation (Fig. 4a, *P*=0.9995) or host cell adhesion (Fig. 4b, *P*=0.8585) relative to the WT strain.

### Motility in strain 1070/13 can be abolished by excision of an IS*1*-adjacent chromosomal region

**Fig. 5.**
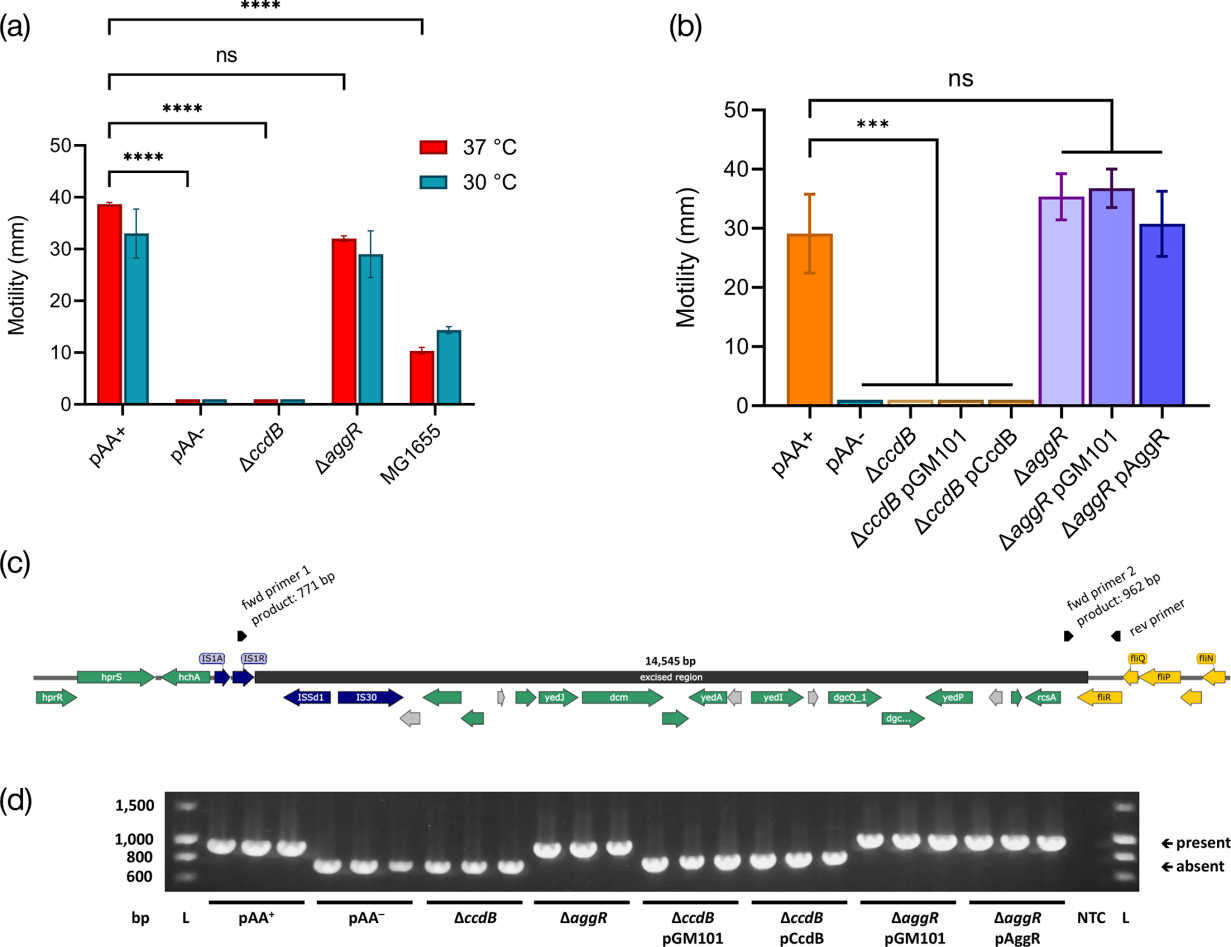
Motility of O104:H4 strain 1070/13. (a) Mean distance from stab point in motility agar at 37 °C (red) and 30 °C (blue). (b) Mean distance from stab point in motility agar at 37 °C with and without the empty vector control (pGM101) or complementation plasmids (pCcdB or pAggR). For both experiments, a lack of evident motility was entered as 1 mm for visualization/analysis purposes. Error bars show sem. Statistical analysis by one- or two-way ANOVA with Tukey’s or Sidak’s multiple comparison tests as appropriate: ns, not significant (*P*≥0.05); ***P<0.001; ****P<0.0001 (from *n*=3 biological replicates). (c) Schematic of 14,545 bp region putatively excised from the 1070/13 chromosome, bordering fliR. Dark blue, transposase-related genes; yellow, flagellar genes; green, other genes of known function; grey, genes encoding hypothetical proteins. PCR primers are indicated by black arrows; rev primer forms a product of either 771 bp (pairing with fwd primer 1, indicating excision of region) or 962 bp (pairing with fwd primer 2, indicating presence of the region). (d) PCR screen of bacteria used in motility assay (three replicates per strain), indicating the presence/absence of the excised region. Representative of multiple independent PCR/motility assays. NTC, no template control; L, ladder. Band sizes (bp) are shown left.

In order to assess the impact of pAA on another putative virulence phenotype, we compared the motility of 1070/13 pAA^–^ with that of the WT strain. The plasmid-free strain was significantly less motile than the WT at both human host (37 °C) and non-host (30 °C) growth temperatures (Fig. 5a, *P*<0.0001). To explore two possible reasons for this, we tested the motility of our Δ*ccdB* mutant as well as a strain lacking the plasmid-encoded regulator AggR. Whilst we observed no motility change in the Δ*aggR* strain (*P*>0.2 for both temperatures), to our surprise, our Δ*ccdB* mutant showed the same lack of motility as the plasmid-free strain (Δ*ccdB* vs. pAA^+^, *P*<0.0001). WT O104:H4 proved significantly more motile than MG1655 regardless of temperature (*P*<0.0001).

Supposing that the CcdAB complex might have a regulatory function that had been disrupted in our mutant, we constructed a complementation plasmid consisting of pGM101_neo_ carrying the whole *ccdAB* operon plus its promoter. We tested motility at 37 °C in strains carrying either this construct or the empty vector (Fig. 5b). The complementation construct was unable to restore motility to the mutant strain (pCcdB vs. pGM101_neo_ in Δ*ccdB* background, *P*>0.9999), suggesting either that the construct somehow failed to compensate for the deletion despite it being a genuine effect or that lack of motility in the Δ*ccdB* mutant is due to an off-target mutation. A similar complementation plasmid carrying *aggR* had no impact on motility (pAggR vs. pGM101_neo_ in Δ*aggR* background, *P*=0.9241).

To confirm the reason for the lack of motility, we analysed the genome sequence of 1070/13 and 1070/13 pAA^–^ strains. This revealed the absence of a chromosomal region bordering IS*1*, IS*3* and IS*30* transposase genes (Fig. 5c) in the plasmid-free strain relative to the parent isolate, resulting in the substitution of the C-terminal 64 aa (~25 %) of flagellar export apparatus component FliR with a nonhomologous sequence of 27 aa. This deletion terminates immediately adjacent to the terminal inverted repeat sequence for IS*1A* as reported by ISFinder (https://www-is.biotoul.fr) [68], suggesting an involvement of this particular transposon in the event. Based on this information, we designed primers (Fig. 5c) to detect the configuration of this genomic region and confirmed that motility loss in all instances, including the Δ*ccdB* mutant, correlated perfectly with the excision of this region. Conversely, strains containing the sequence (and hence the full-length *fliR* gene), including the Δ*aggR* mutant, remained motile. We also noted that the WT isolate sometimes spontaneously became non-motile during laboratory culture and that this had indeed occurred during the growth of the isolate for hybrid genome sequencing – hence, the sequence we provide herein for the 1070/13 parent strain differs from the Illumina data produced by Dallman *et al.* [1] in the absence of the above genomic region. However, in all instances where the strain remained motile throughout a given experiment, PCR confirmed the region’s presence. Representative PCR data of all strains are shown in Fig. 5d. Importantly, we were also able to confirm from our prior assays that this motility defect affects neither biofilm formation nor host cell adhesion (Fig. 4; Δ*ccdB* mutant).

## Discussion

We have shown in this study that the CcdAB TA system on pAA_1070_ is a functional toxin and antitoxin pair, with CcdB likely targeting DNA gyrase, but has only a minor effect on plasmid stability under the conditions tested. We have also shown almost no measurable difference in toxicity between constructs encoding CcdB_101_, the traditionally characterized variant [69,70], and CcdB_110_ (a variant putatively translated from an alternative TTG start codon on pAA_1070_). However, since we saw no difference in toxicity against the native host bacterium, we did not experimentally validate the proteins produced from each construct. It therefore remains possible (even likely) that the TTG start codon is not genuinely recognized by the translation machinery, that it is an annotation error, and that our CcdB_110_ construct is simply producing the CcdB_101_ variant. If ongoing work suggests a potential impact of this alternate start codon, further protein characterization should be performed as a priority.

Divergently transcribed TA systems are not well studied, and given the role of conditional cooperativity, i.e. binding of type II TA complexes to their own promoters, we wondered whether the CcdAB and VapBC systems on pAA_1070_ might impact one another’s gene expression as has been reported for several TA systems [71,73] and the divergently transcribed *par1*/*par2* partitioning loci on pB171 [74]. However, with the finding that this particular VapC variant is non-toxic and that this is likely due to an active site mutation [52], this avenue was not pursued further in this particular work. Nonetheless, regardless of a lack of VapC toxicity, putative regulation by the VapBC complex may also alter the effectiveness of the CcdAB system with regard to plasmid stability. Notably, the strain we used to perform pSTAB analysis does not encode VapBC. Though we did demonstrate a significant *ccdAB*-dependent increase in pSTAB_1070_ stability, the effect was not dramatic (<10-fold). This subtle plasmid-stabilizing role is not atypical of CcdAB TA systems, where some members of this particular TA system family have been shown to impart little or no stabilizing effect overall under laboratory conditions despite being highly toxic [30,31]. It is possible that the pAA CcdAB system functions primarily not as a PSK module but as a regulator of other cellular functions. The regulatory function of both non-toxic and toxic but non-stabilizing TA complexes is currently a subject of ongoing investigation within our laboratory.

We have shown that pAA is a low-copy virulence plasmid, similar to other pathovar-defining plasmids in *E. coli* such as pINV (copy number ~1 per chromosome [47]). We have also confirmed that pAA_1070_ is remarkably stable *in vitro*, with <1% natural plasmid loss occurring over multiple subcultures in liquid media, in agreement with a stress study on the 2011 outbreak strain of hybridized EAEC/EHEC [75] and consistent with our pSTAB results (~0.1% plasmid loss over 20–25 generations of growth on solid medium). The lack of a stabilizing TA system on this plasmid is therefore curious, as such maintenance elements are important for the stability of similar virulence plasmids [76]. We note that the plasmid carries genes related to ParA- and ParM-type partitioning systems; such systems function by physically segregating plasmid molecules during cell division [77] as opposed to the PSK activity of TA systems. TA systems and partitioning systems often work synergistically to optimize plasmid maintenance without excessive PSK [78]. However, the partitioning operons on pAA_1070_ are incomplete and are therefore unlikely to contribute to plasmid maintenance. Instead, as suggested by our pSTAB assay, the dual FII/FIB replicon of pAA_1070_ may be the cause of the plasmid’s *in vitro* stability. Indeed, it was not possible for us to generate pSTAB vectors using the FII replicon alone, contrasting past success with other IncFII and IncA/C plasmids [60,79]. We also note here the work by Zhang *et al.* [50] related to Stx-encoding O104:H4 in case patients, which showed that pAA may be far less stable *in vivo* than *in vitro*. It is possible that laboratory conditions are insufficient to reflect the host environment and that time-dependent pAA loss during infection is a common factor in human EAEC pathogenicity, though this would likely render the bacterium relatively non-pathogenic after passage through the host. Alternatively, pAA loss within the host may be correlated to the production of Shiga toxin. To our knowledge, no research has addressed pAA loss in non-Shigatoxigenic EAEC isolates during the process of infection within patients.

Studies have previously reported the enhanced motility of EAEC strains compared to MG1655 [67], agreeing with our data. The plasmid-encoded EAEC virulence regulator AggR may negatively regulate the aggregation factor antigen 43 [80] and positively regulate flagellar genes [81]. In the former work, no motility effects were observed in the *aggR* mutant background, in agreement with our study. Contrasting this, motility was increased by overexpressing *aggR* in EAEC strain 042 [81]. This did not occur with the addition of our *aggR* complementation vector; though, as the *aggR* promoter is under complex regulation, increasing the gene’s copy number may not increase AggR production per se, or there may be relevant differences between EAEC strains. Notably, EAEC strain 042 carries a larger pAA variant than strain 1070/13 (113.3 kb vs. 75.5 kb) and encodes AAF/II rather than AAF/I [1,14]. Indeed, Prieto *et al.* [81] showed that motility in strain 042 was increased by loss of the pAA plasmid, which we cannot rule out due to conflicting effects of *fliR* mutation in our pAA^–^ strain.

We demonstrated that the loss of motility in EAEC strain 1070/13 can occur readily during laboratory culture and mutagenesis. This phenotype was found to correlate perfectly with the spontaneous excision of a chromosomal region containing the 3′ end of the *fliR* gene, bordered by transposase genes and terminating immediately adjacent to the *fliR*-proximal IS*1A* terminal inverted repeat. Such deletions occur readily via IS*1* variants [82,84]. The same chromosomal region, including IS elements, is present in the 2011 outbreak strain TY2482 (shotgun sequence scaffold4; accession NZ_GL989603.1) [6], though it is unknown if motility loss has ever occurred during experiments with that strain. We have also conducted further blast analysis of fully assembled genome sequences within GenBank, finding that this particular *fliR*-IS*1* architecture seems to be predominantly restricted to the O104:H4 serotype. This includes historical strains isolated prior to the major 2011 outbreak, e.g. a South Korean strain isolated in 2001 (accession number CP009050.1) and strain 55989 isolated pre-2000 from the Central African Republic (accession number CU928145.2). We also found the same sequence in O181:H4 isolates (accession numbers CP096976.1 and CP086259.1), which are associated with haemolytic uremic syndrome and are likely the result of O-antigen diversification amongst Stx-positive strains [85]. However, we found no evidence within the assembled sequence database of the *fliR*-disabling excision having occurred in strains other than 1070/13. The excision may therefore result from adaption to the non-host environment. Indeed, motility loss via a different IS*1*-mediated flagellar gene deletion has been reported in *E. coli* K-12 MG1655 as an adaption to laboratory culture [86]. We report our findings as a cautionary tale in the era of readily accessible whole-genome sequencing; knowledge of the excised region in advance of our motility and complementation experiments would have been an advantage.

Whilst reversible motility loss via transposon excision and re-insertion may reflect a type of phase variation in EAEC, and phenotypic reversions of IS*1*-mediated resistance gene deletions have been detected on plasmid R100 [87], we did not observe reversion to a motile phenotype during our assays. Alternatively, the ability of the organism to lose motility may confer an evolutionary benefit and future route to heightened pathogenesis, as some other enteric pathogens such as *Shigella* contain cryptic flagellar genes [88]. The production of AAF in EAEC may even directly encourage loss of motility, as other chaperone-usher fimbriae are known to downregulate flagellum-mediated motility in uropathogenic *E. coli* [89]; a ‘stick or swim’ outcome. We also note that the excised region contains other important genes such as *dcm*, encoding DNA-cytosine methyltransferase, and *rcsA*, implicated in multiple phenotypes including *Salmonella* motility [90]. Interestingly and in keeping with the idea of an evolutionary route, *rcsA* is pseudogenized in *Yersinia pestis* as a pathoadaption to enable higher biofilm formation and hence greater transmission from the insect vector [91]. However, in *E. coli*, RcsA was instead characterized as a positive regulator of capsular polysaccharide production [92]. We ultimately observed no impact of the loss of this genetic region on either biofilm formation or host cell adhesion in our experiments. Indeed, we found that the biofilm formation of 1070/13 was poor regardless of either plasmid loss or transposon excision, significantly lower than EAEC 042 and instead similar to non-pathogenic strain MG1655.

Our study reflects the complexity of working with pathogenic outbreak isolates rather than laboratory strains and validates the importance of doing so. We have reported the influence of distinct MGEs (both plasmid and transposon) in the virulence-related phenotypes of EAEC, contributing to the understanding of this neglected enteric pathogen in its own right.

## supplementary material

10.1099/mic.0.001550 Uncited Supplementary Material 1.

## Abbreviations

AAF: aggregative adherence fimbriae
DAP: diaminopimelic acid
ddPCR: digital droplet PCR
DMEM: Dulbecco’s modified Eagle’s medium
EAEC: enteroaggregative *Escherichia coli*
EHEC: enterohaemorrhagic *Escherichia coli*
FAM: fluorescein
HEX: hexachlorofluorescein
IS: insertion sequence
LB: lysogeny broth
MGE: mobile genetic element
pAA: aggregative adherence plasmid
pINV: invasion plasmid
PSK: post-segregational killing
TA: toxin–antitoxin
YESCA: yeast extract casamino acids
