# Supplementary material for: Genetic and phenotypic analysis of the virulence plasmid of a non-Shigatoxigenic enteroaggregative Escherichia coli O104:H4 outbreak strain
Source: Microbiology (Reading). 2025 Mar 27;171(3):001550. doi: 10.1099/mic.0.001550 (PMC11950199; doi:10.1099/mic.0.001550)
Supplement: Uncited Supplementary Material 1. [file mic-171-01550-s001.pdf]

Supplementary Table 1: Bacterial strains used in this study.

Nomenclature	Species/serotype/genotype	Source
042	Prototypical enteroaggregative *E. coli* strain (O44:H18)	M. D. Goldberg (BCU) [14]
1070/13	Enteroaggregative *E. coli* O104:H4 strain 1070/13	[1]
1070/13 Δ*aggR*	EAEC 1070/13, Δ*aggR*::*cat*	This study
1070/13 Δ*ccdB*	EAEC 1070/13, Δ*ccdB*::*cat*	This study
1070/13 pAA^-	Plasmid-free derivative of EAEC 1070/13	This study
ccdB Survival 2 $T1^R$	*E. coli* CcdB-resistant cloning strain (genotype unknown)	Invitrogen
DB3.1	*E. coli* CcdB-resistant cloning strain (*gyrA462*)	Invitrogen
MFD*pir* Δ*hsdR*	*E. coli* MFD*pir* Δ*hsdR;* conjugation donor	C. M. Tang (Oxford)
MG1655	*E. coli* K-12 MG1655 wild type	[45]
PAO1	*P. aeruginosa* PAO1 wild type	[64]

Supplementary Table 2: Plasmids used in this study.

Plasmid ID	Construction/purpose	Source
pAggR	$pGM101_{neo}$ containing entire *aggR* locus	This study
pBAD33	Arabinose-inducible vector for toxicity assays	[53]
pBAD33::$ccdB_{110}$	pBAD33 encoding $CcdB_{110}$	This study
pBAD33::$ccdB_{101}$	pBAD33 encoding $CcdB_{101}$	This study
pBAD33::$ccdB_{M90T}$	pBAD33 encoding $CcdB_{M90T}$	This study
pBAD33::*relE1*	pBAD33 encoding RelE/ParE 1	This study
pBAD33::*relE2*	pBAD33 encoding RelE/ParE 2	This study
pBAD33::$vapC_{142}$	pBAD33 encoding $VapC_{142}$	This study
pBAD33::$vapC_{133}$	pBAD33 encoding $VapC_{133}$	This study
pCcdB	$pGM101_{neo}$ containing entire *ccdAB* locus	This study
pCONJ5K	Mobilisable vector for mutagenesis	[34]
pGM101	Promoterless vector for antitoxicity assays	[30]
$pGM101_{neo}$	Neomycin-resistant variant of pGM101	This study
$pGM101_{neo}$::*ccdA*	$pGM101_{neo}$ containing *ccdA* plus its promoter	This study
pIB279	*sacB*-Neo^R cassette for pSTAB vectors	[58]
pMW_O104	pSTAB with FII/FIB replicons from pAA_{1070}	This study
pMW_O104::*ccdAB*	pMW_O104 containing entire *ccdAB* locus	This study
pUC19	High-copy cloning vector	New England Biolabs

Supplementary Table 3: Oligonucleotide primers used in this study.

Primer ID	Sequence (5’-3’)	Target
GMntu106	gcgcatcccagagggacatcttatattccccagaacatcaggttaatg	*ccdAB* for pSTAB
GMntu107	gtgatgggttaaaaaggatccggatctgccggaaatgg	
GMntu112	gcgcatcccagagggacatcgatcctttttaacccatcacatatacctgccgttcac	*sacB-NeoR*
GMntu113	gttaaaaaggatcgatgtccctctgggatgcgctccggatgaatatgatgatc	pAA_{1070} replicon
GMntu114	caggacgacgaggcttgcaccttcataatcggtagtgtatgctgtttttctgg	
GMntu115	gcatacactaccgattatgaaggtgcaagcctcgtcgtcctggccggaccacgctatctg	*sacB-NeoR*
GMntu132	ccatttccggcagatccggatcctttttaacccatcacatatacctgccgttcac	pMW_O104 for
GMntu133	ctggggaatataagatgtccctctgggatgcgctccggatgaatatgatgatc	*ccdAB* insertion
MS123	cgaagcggcatgcatttacg	pGM101 for
MS124	ccttcgcgcgcgaattgatc	complementation
MS125	gatcaattcgcgcgcgaagggactgggctgcaattaag	*aggR* for
MS126	cgtaaatgcatgccgcttcgtcattggcttttaaaataagtc	complementation
MS127	gatcaattcgcgcgcgaaggtttcctcagtgtggtacac	*ccdAB* for
MS128	cgtaaatgcatgccgcttcgttatattccccagaacatcag	complementation
RWntu011	ccgggtaccgagctcgaattc	pBAD33
RWntu012	ggatcctctagagtcgac	
RWntu013	aattcgagctcggtacccggcgacgaaggaagatttgac	*vapC*$_{142}$
RWntu014	aggtcgactctagaggatccttacttcacccagtcttc	
RWntu015	aattcgagctcggtacccgggacccgaacgaagacctatatg	*vapC*$_{133}$
RWntu016	aggtcgactctagaggatccttacttcacccagtcttcc	
RWntu017	aattcgagctcggtacccggttattgaaatgaacggctc	*ccdB*$_{110}$
RWntu018	aggtcgactctagaggatccttatattccccagaacatcag	
RWntu028	agaaacgcaaaaaggccatc	pGM101 (to make
RWntu029	ctgtcagaccaagtttactc	$pGM101_{neo}$)
RWntu030	gatggcctttttgcgtttctcttcacgctgccgcaagc	*neoR*
RWntu031	gagtaaacttggtctgacagaggcggcggtggaatcga	
RWntu040	tcgcgcgcgaaggcggatctgccggaaatgg	*ccdA*
RWntu041	gcatgccgcttcgtcaccagtccctgttctc	
RWntu074	aggtcgactctagaggatccttatattccccagaacatcagg	*ccdB*$_{101}$
RWntu075	aattcgagctcggtacccggttgccgatgagaacaggg	
RWntu081	aattcgagctcggtacccggaaatgaacggctcttttg	*ccdB*$_{M90T}$
RWntu082	aggtcgactctagaggatccttatattccccagaacatcag	
RWntu088	aattcgagctcggtacccggactgacaatccgttaccg	*relE/parE 1*
RWntu089	aggtcgactctagaggatccctacctgttttctcgtgtg	
RWntu090	aattcgagctcggtacccggaagctggcgcgagggctt	*relE/parE 2*
RWntu091	aggtcgactctagaggatcctcagttgaaatgacggctggcatc	
RWntu106	tcgtttgaaatcccgcttcg	*dnaA* (ddPCR)
RWntu107	cgttttcgtcggcctttttc	
RWntu108	agccgatccgttaaaagcac	*fliC* (ddPCR)
RWntu109	tggtggtgttgttcaggttg	
RWntu110	tgttaacgaacgtcgcgaag	*hns* (ddPCR)
RWntu111	attgctgcagcttacgagtg	
RWntu112	gctgtaggacccacttattagc	*aggA* (ddPCR)
RWntu113	tcctccacaaactgttggtg	
RWntu114	atatcggtggtcatcatgcg	*ccdB* (ddPCR)
RWntu115	aagtctcccgtgaactttaccc	
RWntu116	gcaatagccaatttgcacat	*wzy* (ddPCR)
RWntu117	cccggggcaattatcattaa	
RWntu140	ggtacccggggatcctctag	pUC19
RWntu141	gagctcgaattcactggcc	
RWntu142	cggccagtgaattcgagctccagtgttgcaaaaatggtg	*aggR* downstream
RWntu143	cagcctacacttgacttattttaaaagccaatg	

Supplementary Table 3 cont.: Oligonucleotide primers used in this study.

Primer ID	Sequence (5'-3')	Target
RWntu144	aataagtcaagtgtaggctggagctgcttc	*cat* (Δ*aggR*)
RWntu145	tgataaagacatgggaattagccatggtcc	
RWntu146	taattcccatgtctttatcaggcaactctgc	*aggR* upstream
RWntu147	ctagaggatccccgggtaccggcgcgcatcctgtatattattg	
RWntu148	cagcctacacctgatgttctggggaatataaatgtc	*ccdB* downstream
RWntu149	cggccagtgaattcgagctcgcgctttctctgtcctgc	
RWntu150	tggtgaaatgatgggaattagccatggtcc	*cat* (Δ*ccdB*)
RWntu151	agaacatcaggtgtaggctggagctgcttc	
RWntu152	ctagaggatccccgggtaccctccctgacctgtgatgc	*ccdB* upstream
RWntu153	taattcccatcatttcaccagtccctgttc	
RWntu154	aacgacggccagtgtttaaaggcgcgcatcctgtatattattg	*aggR* (deletion)
RWntu155	agcaggaaacagctatgacgcagtgttgcaaaaatggtgg	
RWntu156	cgtcatagctgtttcctg	pCONJ5K
RWntu157	tttaaacactggccgtcg	
RWntu158	agcaggaaacagctatgacggcgctttctctgtcctgc	*ccdB* (deletion)
RWntu159	aacgacggccagtgtttaaactccctgacctgtgatgc	

Supplementary Table 4: ddPCR master mix.

Component	Concentration or quantity per 20 µl reaction
2x ddPCR Supermix for Probes (No dUTP)	10 µl
Target primer/probe mix (FAM)	900 nM primers/250 nM probes
Reference primer/probe mix (HEX)	900 nM primers/250 nM probes
DNA	50 ng

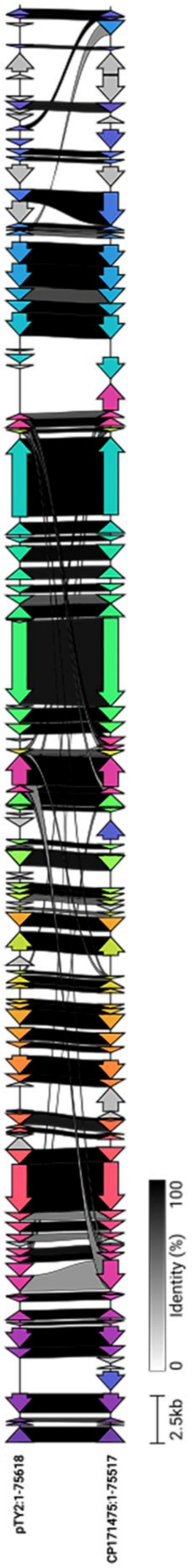

Supplementary Figure 1: Comparative analysis of linear plasmid maps pTY2 and pAA_{1070} (CP171475). Colours represent homologous CDSs. Homologous regions shared between the two plasmids are shaded in grey according to percentage nucleotide identity greater than 70 %. Image produced using clinker gene cluster comparison [42] (https://cagecat.bioinformatics.nl/).

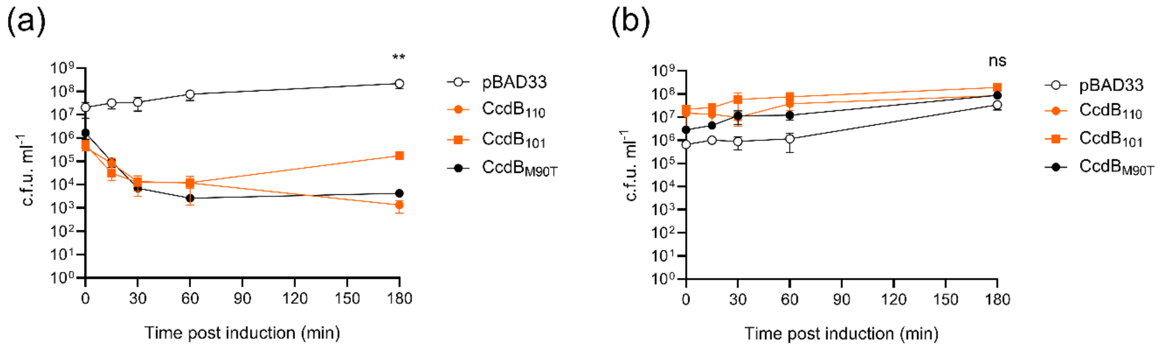

Supplementary Figure 2: Toxicity of CcdB from pAA$_{1070}$ in CcdB-resistant cloning strains. Mean bacterial viability after induction of toxin production from pBAD33; (a) in *E. coli ccdB* Survival 2 T1^R; (b) in *E. coli* gyrase mutant DB3.1. Empty vector control in each experiment is indicated as pBAD33. Error bars show standard error of the mean. Statistical analysis by two-way ANOVA with Dunnett's multiple comparison tests: ns, not significant ($P > 0.05$); ** $P < 0.01$ (from n = 3 biological replicates).

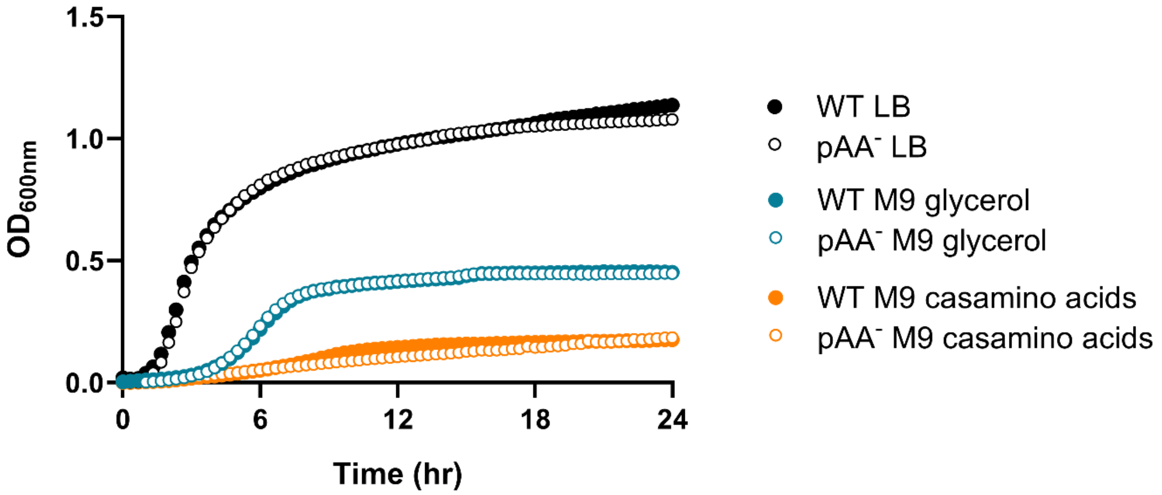

Supplementary Figure 3: Growth of O104:H4 strain 1070/13 (WT) and its plasmid-free derivative (pAA$^-$) in various laboratory media. OD_{600nm} measured every 20 minutes at 37 °C with aeration in 24-well plate. Mean of two biological replicates shown (from six technical replicates).

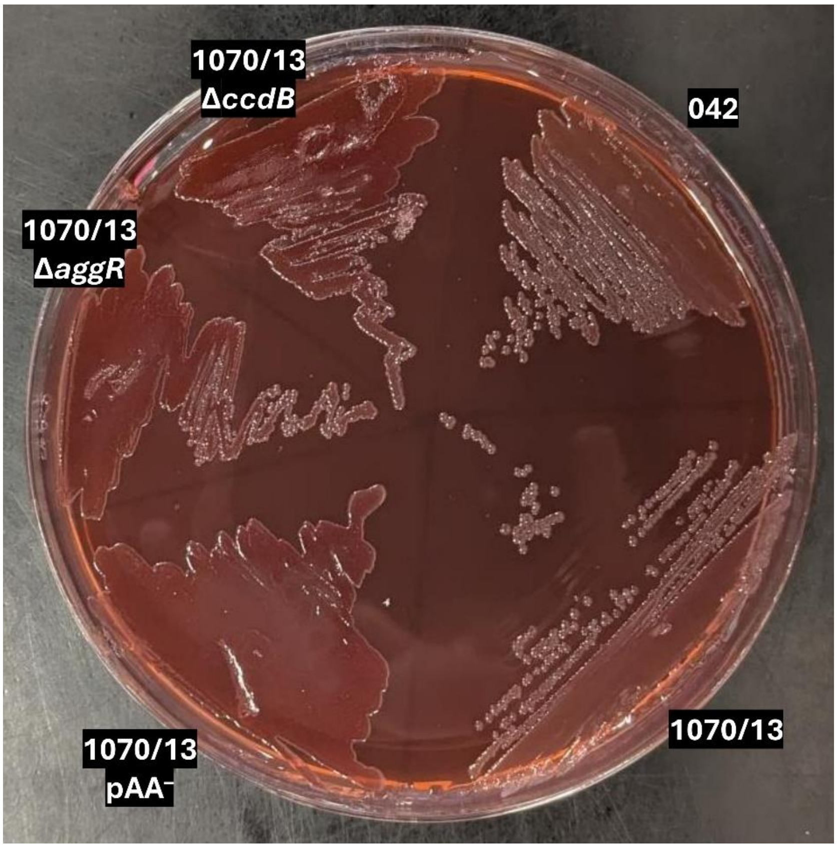

Supplementary Figure 4: Growth of O104:H4 strain 1070/13 and its derivatives on YESCA medium supplemented with Congo red dye. Plate incubated at 37 °C.

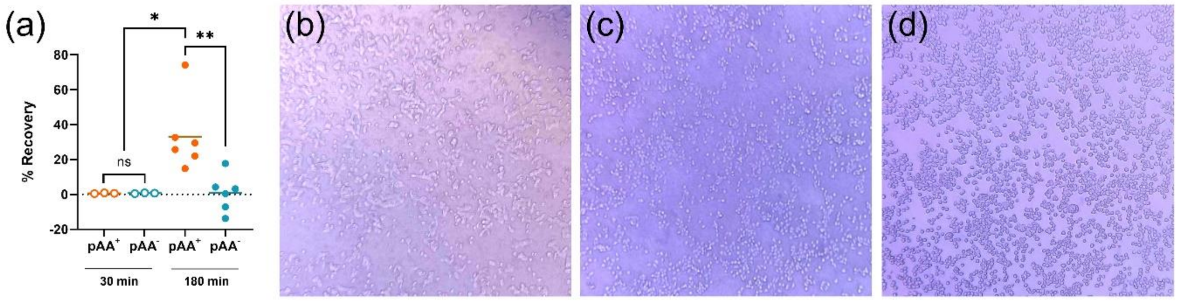

Supplementary Figure 5: Effect of pAA on adherence of O104:H4 strain 1070/13 to HT-29 cells. (a) Per cent recovery of bacterial cells (normalised to inoculum) after incubation for either 30 min (empty circles) or 180 min (solid circles) with HT-29 cells. Each point is one biological replicate. Solid lines show the mean. Statistical analysis by one-way ANOVA with Tukey's multiple comparison tests: ns, not significant ($P \geq 0.05$); * $P < 0.05$; ** $P < 0.01$ (from $n \geq 3$ biological replicates). (b-d) Light microscopy at 40x magnification of HT-29 cells at 180 min post-infection with either (b) 1070/13, (c) 1070/13 pAA⁻ or (d) no bacteria.
